# Predictive value of node-RADS scoring system for axillary lymph node metastasis in breast cancer

**DOI:** 10.1186/s12880-026-02181-x

**Published:** 2026-01-28

**Authors:** Hazal Selvi Çubuk, Murathan Erkent, Burak Yağdıran, Sedat Yıldırım

**Affiliations:** 1 Department of Radiology, Faculty of Medicine, Baskent University, Yukarı Bahcelievler, Mareşal Fevzi Cakmak Street. No:45, Ankara, Cankaya 06490 Türkiye; 2 Department of General Surgery, Faculty of Medicine, Baskent University, Yukarı Bahcelievler, No:45, Ankara, Cankaya 06490 Türkiye

**Keywords:** Axillary lymph node, Node-RADS, Magnetic resonance imaging, Extranodal extension, Breast cancer

## Abstract

**Background:**

We aimed to evaluate the predictive value of preoperative Node-RADS determination at imaging for axillary lymph node (ALN) involvement in cases of breast cancer.

**Materials and methods:**

Node-RADS was determined in all cases using preoperative breast magnetic resonance imaging (MRI) by two radiologists blinded to pathologic results. The ROC curves and AUCs depicted the overall diagnostic performance of the Node-RADS score for lymph node involvement. In addition, the presence of a flare-like perinodal signal was also evaluated as an imaging feature suggestive of extranodal extension. Size and morphological parameters were assessed separately for their association with metastatic involvement.

**Results:**

Both readers demonstrated high diagnostic accuracy in predicting ALN metastasis using the Node-RADS system, with the best diagnostic performance observed at a cutoff value above Node-RADS 2. In logistic regression analysis, heterogeneous internal texture of the lymph node and flare sign were also found to be statistically significantly associated with invasion (*p* < 0.05). The presence of a fatty hilum was a statistically significant predictor (*p* < 0.001) associated with a markedly lower likelihood of lymph node metastasis (OR = 0.019, 95% CI = 0.002–0.153).

**Conclusion:**

The Node-RADS scoring system demonstrates high diagnostic reliability and reproducibility in the evaluation of axillary lymph nodes in breast cancer. However, the size criterion for ALN assessment may need to be re-evaluated, and the inclusion of size or extranodal extension parameters in the scoring system should be reconsidered.

## Introduction

Lymph node metastasis is a key determinant in the TNM staging system for breast cancer and remains one of the most important prognostic indicators [1, 2]. Although histopathologic evaluation continues to serve as the diagnostic gold standard, reliable preoperative characterization of axillary lymph nodes (ALNs) contributes substantially to clinical decision-making and surgical planning. The Node Reporting and Data System (Node-RADS) was introduced to provide a unified and reproducible framework for assessing lymph node size, morphology and configuration across different malignancies [3]. Node-RADS has demonstrated moderate sensitivity and high specificity for detecting LN invasion across various cancers [4]. Numerous radiologic studies have focused on defining the imaging features associated with metastatic axillary nodal involvement in breast cancer [5–8]. Early applications in prostate, bladder, lung, colorectal, and gastric cancers have shown that Node-RADS offers moderate sensitivity and high specificity in predicting nodal invasion [9–13]. However, there are limited studies investigating its use in the assessment of ALN metastasis and preoperative staging with dynamic breast MRI breast cancer [14, 15]. To address this gap in the literature, our study aims to evaluate the predictive value of the Node-RADS scoring system for assessing ALN invasion in breast cancer. This gap is notable, given the increasing clinical reliance on MRI-based axillary staging and the known variability associated with radiologist experience, nodal size thresholds, and interpretive criteria [4, 8]. The aim of this study was to evaluate the diagnostic performance of the Node-RADS scoring system for assessing axillary lymph node invasion in breast cancer, with the goal of reducing unnecessary axillary biopsies. Specifically, we sought to assess the predictive value of each individual Node-RADS criterion, including lymph node size and configurational features, in distinguishing metastatic from benign nodes. In addition, we investigated whether ancillary findings, such as radiologic evidence of the flare sign indicative of extranodal extension, were associated with a higher likelihood of nodal invasion.

## Materials and methods

Following approval by the Baskent University Medical and Health Sciences Research Board (KA25/257), patients diagnosed with breast cancer by histopathology between 2013 and 2024 were included in our study. Eligible patients were those who underwent preoperative dynamic breast MRI followed by surgical treatment with either segmental or other types of mastectomy, in conjunction with axillary dissection or sentinel lymph node biopsy. The histopathological outcome of the lymph nodes was accepted as the gold standard. In cases of unilateral breast cancer in the analysis, only the ALN ipsilateral to the breast tumor were included. Patients with bilateral tumors in whom an index side could not be defined were excluded from the study. Patients without preoperative dynamic breast MRI, those who had received neoadjuvant chemotherapy, or those with insufficient pathological results were also excluded from the study. All ALNs obtained through sentinel lymph node biopsy or axillary lymph node dissection were evaluated by pathologists. The specimens were fixed in formalin, serially sectioned, and examined on hematoxylin–eosin–stained (H&E) slides at multiple levels. When micrometastatic disease was suspected or when routine H&E evaluation was inconclusive, additional immunohistochemical staining was performed. The classification of metastatic deposits followed the American Joint Committee on Cancer (AJCC) 8th Edition definitions: macrometastasis is defined as a metastatic focus larger than 2 mm; micrometastasis is defined as deposits measuring more than 0.2 mm and up to 2 mm; and isolated tumor cells are defined as clusters measuring 0.2 mm or less that are not categorized as metastatic involvement. These criteria were consistently applied throughout the pathological assessment.

**Fig. 1:**
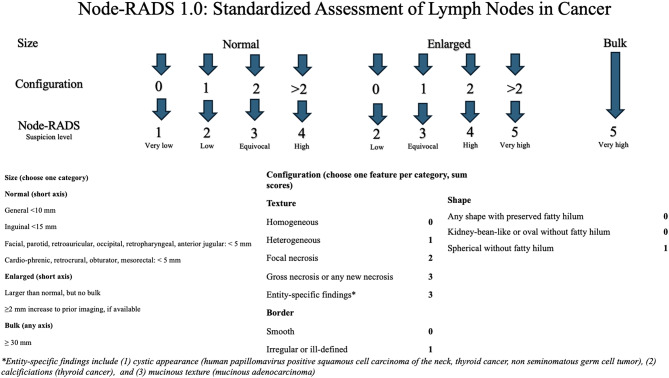
Node-RADS flowchart with a brief description of the criteria for lymph node assessment

In this retrospective design, patient data, including age and family history, were retrieved from medical records. Pathology reports were reviewed for tumor histologic subtype, receptor status, Ki-67 index, and lymph node invasion. The total number of lymph nodes removed, pathologic T stage (pT), N stage (pN), and tumor grade based on the Nottingham Histologic Score were recorded. Preoperative dynamic breast MRI examinations were independently evaluated by two radiologists with more than 5 years of experience and one with 3 years of subspecialty practice in breast imaging. Both were blinded to the clinical and pathological data. In dynamic contrast-enhanced MRI (DCE-MRI), Short Tau Inversion Recovery (STIR), fat-suppressed T1-weighted, and subtraction sequences were analyzed to assess tumor size, tumor to skin distance, number of foci, quadrant location of the index lesion, and lesion margins. For each lymph node located on the same side as the primary tumor, size, cortical thickness, shape, border, internal texture, presence of necrosis and presence of a fatty hilum were assessed. The DCE-MRI assessment included evaluation categories based on size and morphological criteria. In addition, the flare sign indicative of extranodal extension was assessed on STIR sequences. Node-RADS scoring was performed according to previously published criteria [3]. Each lymph node was evaluated based on short-axis diameter using 10 mm as the threshold for enlargement, overall shape (oval vs. round), margin definition (smooth vs. irregular), presence or absence of fatty hilum, internal texture (homogeneous vs. heterogeneous). and necrosis. Scores ranged from 1 to 5, with 1 indicating a very low likelihood and 5 indicating a very high likelihood of metastatic involvement. A schematic overview of the scoring criteria is presented in Fig. 1. In the present study, all ALNs on the same side of the breast mass were evaluated rather than selecting only the node with the largest short-axis diameter. Subsequently, the lymph node with the highest Node-RADS score was chosen for analysis, ensuring that the most suspicious node, rather than the largest, was included in the evaluation. Extranodal extension, which is not included in the Node-RADS scoring system, was also evaluated. Lymph nodes were anatomically classified as axillary, supraclavicular, or intramammary [16]. All patients were examined using DCE-MRI performed with 1.5-T MR (Siemens Magnetom Avanto, Symphony, Erlangen, Germany). A dedicated double breast coil was used with the patient in prone position. All examinations were performed at a single institution using a standardized breast MRI protocol that remained consistent throughout the study period. The same 1.5-T MRI system protocol, breast coil type, and dynamic contrast-enhanced (DCE) sequence parameters were used for all patients.

Standard protocols for breast imaging, including, precontrast axial, nonfat suppressed, T1-weighted (repetition time/echo time [TR/TE], 450/9.6; matrix, 257 × 384; number of excitation [NEX], 2; slice thickness, 4 mm) and STIR (TR/TE, 4860/69; matrix, 314 × 320; NEX, 2; slice thickness, 3.5 mm) sequences were performed. Both before and after intravenous contrast material injection, six sequential fat-suppressed three-dimensional (3-D) T1-weighted sequences were obtained, and subtraction was performed. A bolus dose of contrast was injected intravenously at a dose of 0.1 mmol/kg of body weight. The contrast agent dose (0.1 mmol/kg) was constant, and the contrast injection rate followed routine departmental practice (approximately 2 mL/s) with no major variations throughout the study period. In all MRI examinations, a gadolinium-based contrast agent was administered intravenously, using either gadoterate meglumine or gadobutrol at a standard dose of 0.1 mmol/kg, depending on institutional availability.

The scanning parameters for DCE-MRI were TR/TE, 4.43/1.73; matrix, 336 × 448; NEX, 1; slice thickness, 1.2 mm; flip angle, 10°; field of view, 34 × 34 cm.

Statistical analyses were performed using SPSS software (version 22.0, SPSS Inc.), and ROC analysis was conducted with MedCalc version 22.014. The Kolmogorov–Smirnov test was applied to assess normality. Continuous variables with normal distribution were expressed as mean ± standard deviation, while non-normally distributed variables were presented as median (IQR, Q1–Q3). Categorical variables were summarized as frequencies (n) and percentages (%). Comparisons of categorical variables were performed using the Pearson Chi-square test or Fisher’s exact test, as appropriate. For the comparison of the two independent groups, the Student’s t-test was used for normally distributed variables, whereas the Mann–Whitney U test was applied for non-normally distributed variables. Spearman Correlation test was applied for correlation analysis. Logistic regression analysis was performed to evaluate the effect of potential risk factors. A p-value of < 0.05 was considered statistically significant.

## Results

A total of 83 female patients with breast cancer who underwent CE-MRI between 2013 and 2024 were included in the study. The mean age of the patients was 56.51 ± 13.86 years (range, 25–96 years). Regarding hormone receptor status, estrogen receptor (ER) positivity was observed in 77.1% of cases, progesterone receptor (PR) positivity in 65.1%, and c-ERB2 positivity in 34.9%. Histopathological analysis revealed that the majority of patients had invasive carcinoma (73.5%), whereas ductal carcinoma in situ (DCIS; 4.8%), invasive lobular carcinoma (8.4%), mixed carcinoma (7.2%), and other tumor subtypes (6.0%) were less frequently reported. A family history of breast cancer was identified in 6% of patients. ALN metastases was positive in 42.2% and negative in 57.8% of the cohort (Table 1).

**Table 1:** Demographic and clinicopathological characteristics of the patients (*n* = 83)

Variables	Values
**Age (years)**, Mean ± SD (min–max)	56.51 ± 13.86 (25.0–96.0)
	**n (%)**
**ER (+)**	64 (%77.1)
**PR (+)**	54 (%65.1)
**c-ERB2 (+)**	29 (%34.9)
**Histologic subtypes of BC**	
DCIS	4 (%4.8)
Invasive Carcinoma	61 (%73.5)
Lobular Carcinoma	7 (%8.4)
Mixt type	6 (%7.2)
Other	5 (%6.0)
**Family History**	
Negative	78 (%94)
Positive	5 (%6)
**Axillary lymph node metastasis**	
Negative	48 (%57.8)
Positive	35 (%42.2)

**Fig. 2:**
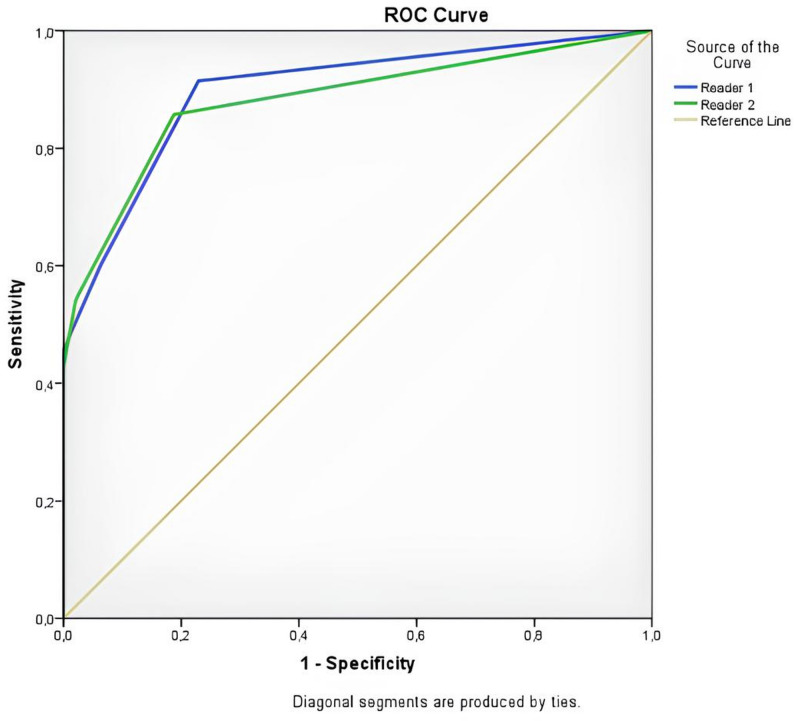
ROC curve analysis of Node-RADS for predicting axillary lymph node metastasis

According to ROC curve analysis, the Node-RADS score demonstrated a strong ability to predict ALN metastasis (ALNM). For Reader 1, the AUC was 0.897 (95% CI: 0.826–0.968), with a sensitivity of 91.4% and a specificity of 77.1% at the cutoff value of Node-RADS > 2 (*p* < 0.001). For Reader 2, the AUC was 0.837 (95% CI: 0.739–0.909), yielding a sensitivity of 77.1% and a specificity of 81.5% (*p* < 0.001). These findings indicate that Node-RADS provides a reliable diagnostic performance across readers, although predictive accuracy may vary with reader experience (Fig. 2; Table 2).

**Table 2:** Diagnostic performance of Node-RADS for axillary lymph node metastasis prediction

	AUC^*^ (95% Cl)	Cutoff	*p*	Sensitivity(%)	Specificity(%)
Reader_1	0.897 (0.826–0.968)	> 2	< 0.001	91.4	77.1
Reader_2	0.837 (0.739–0.909)	> 2	< 0.001	77.1	81.5

^*^AUC; Area Under the Curve

**Fig. 3:**
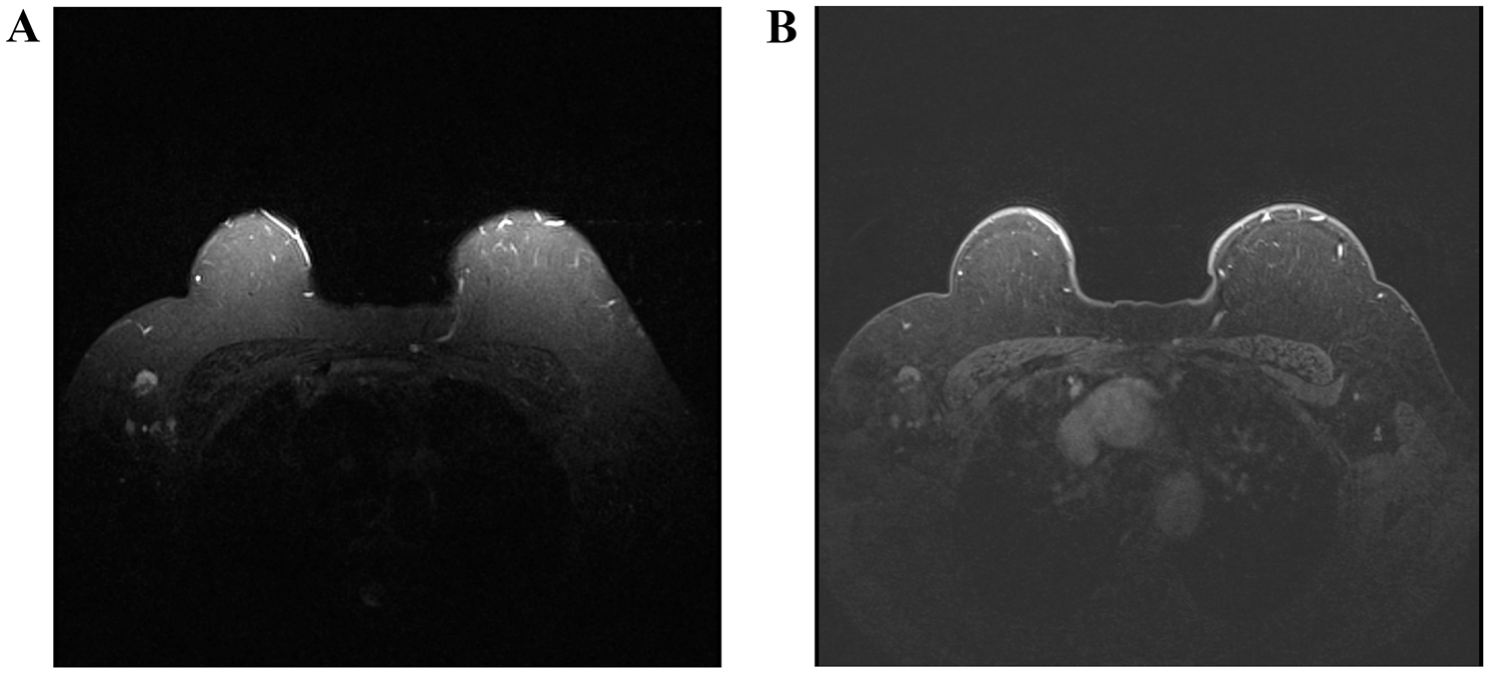
**A** On axial STIR image demonstrates a spherical lymph node with a preserved fatty hilum. **B** On post-contrast T1-weighted fat-suppressed imaging, heterogeneous cortical enhancement is observed. The lymph node was classified as Node-RADS category 3, and histopathological analysis confirmed metastatic involvement

**Fig. 4:**
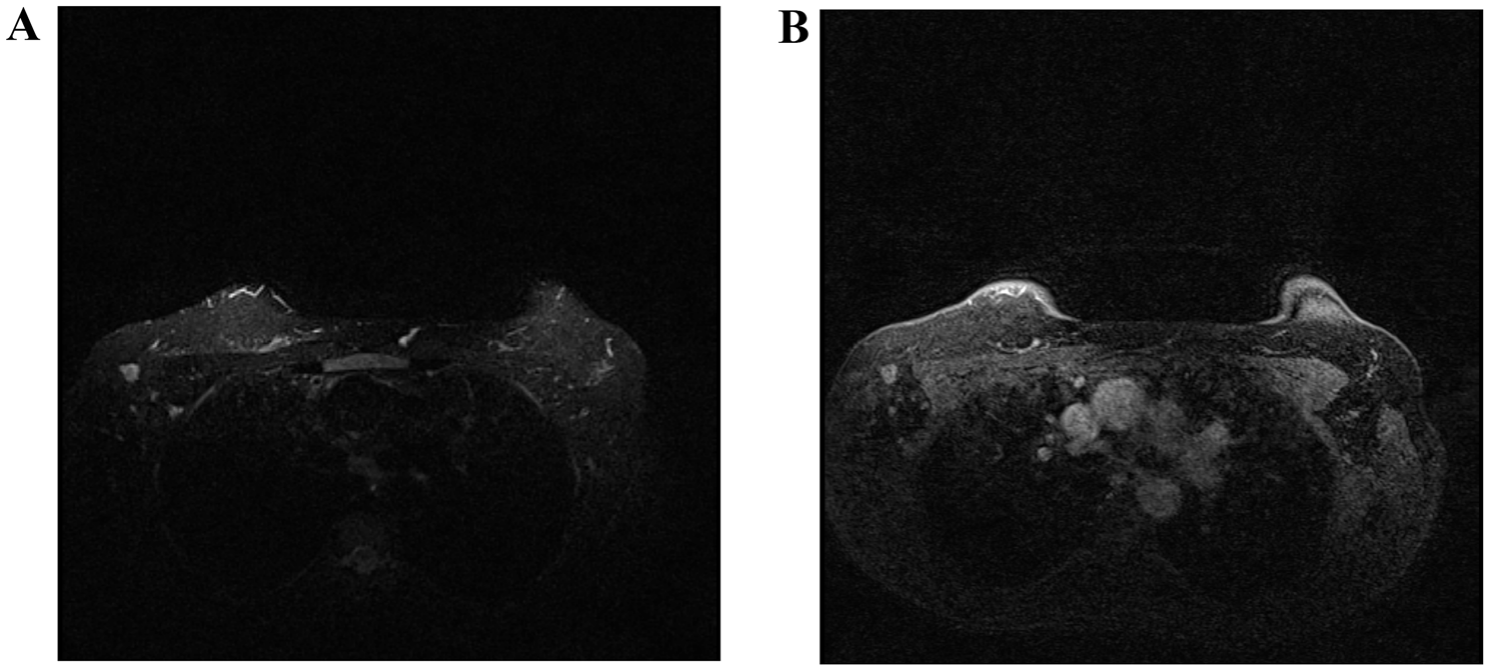
**A** On axial STIR MR imaging, a normal size round lymph node with irregular margins is demonstrated. **B** Post-contrast T1-weighted fat-suppressed images reveal heterogeneous cortical enhancement, and the lymph node is classified as Node-RADS category 4 and histopathological analysis confirmed metastatic involvement

**Fig. 5:**
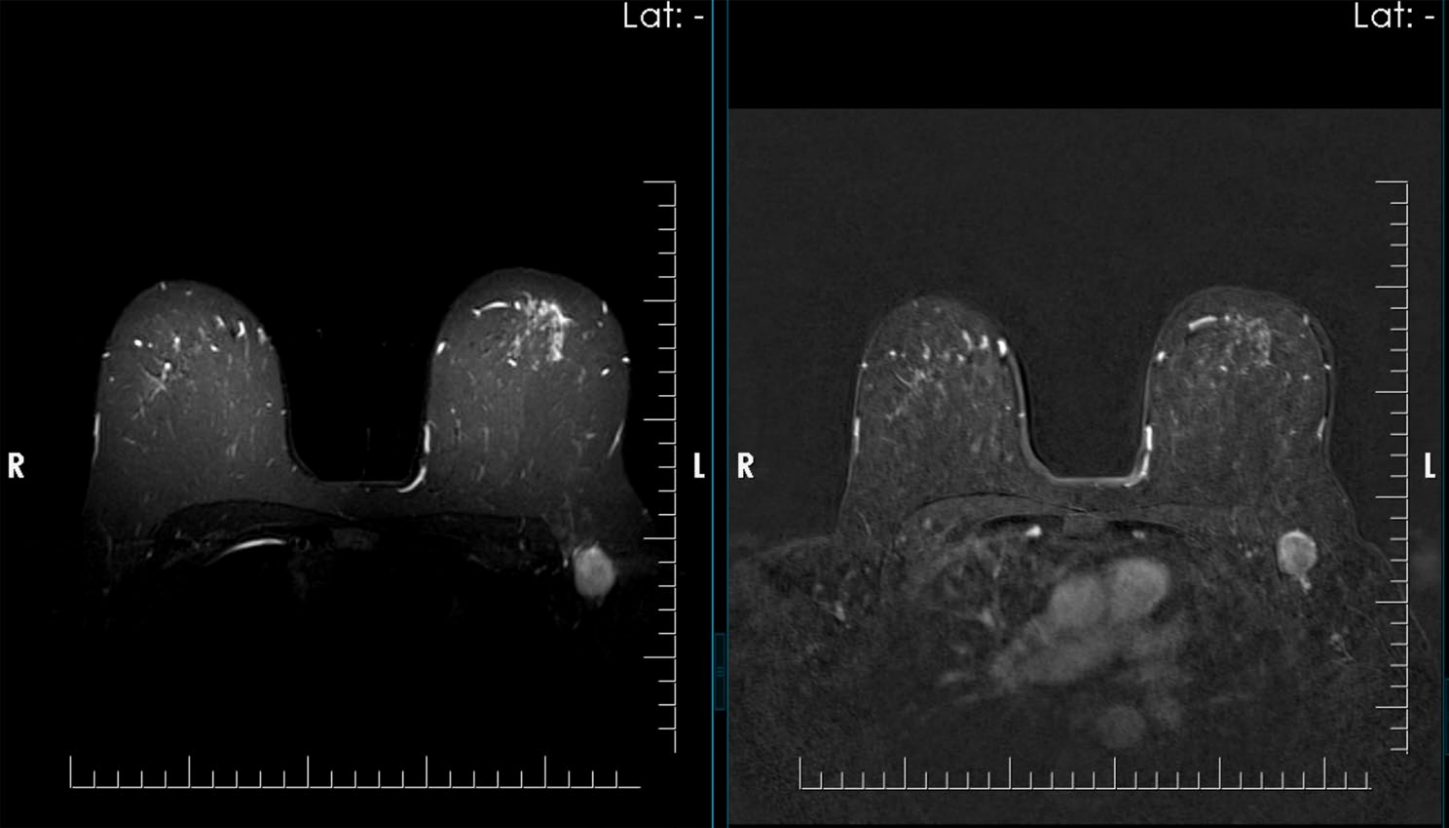
On the left, axial STIR MR images of the left axilla demonstrate an enlarged, spherical lymph node with a heterogeneous cortex and absence of a fatty hilum. On the right, post-contrast Dixon fat-suppressed images show heterogeneous enhancement. The Node-RADS category was 5, and histopathologic examination confirmed metastasis

According to ROC analysis, specificity and PPV increased significantly with increasing Node-RADS scores for both readers, while sensitivity and NPV decreased gradually (Table 3). Youden index and likelihood ratio (LR) analyses were calculated by comparing different threshold values. For Reader 1, the threshold with the highest Youden index was Node-RADS > 1 (J = 0.685), with sensitivity of 91.4% and specificity of 77.1%. A more balanced distribution was observed at a threshold value of Node-RADS > 2 (J = 0.538; sensitivity of 60.0% and specificity of 93.75%; LR + = 9.60). A similar trend was observed in Reader 2, with the highest Youden index calculated at Node-RADS > 1 (J = 0.584). Clinically, more balanced performance was achieved at a threshold value of Node-RADS > 2 (J = 0.494) (sensitivity 51.4%; specificity 97.92%; LR + = 24.73). Although a threshold value of > 1 was advantageous in terms of sensitivity when all these results were evaluated together, Node-RADS > 2 was found to be more appropriate because it provided high LR + in both readers (9.60 and 24.73) and in terms of the balance between sensitivity and specificity. Therefore, the optimal diagnostic threshold in this study was defined as Node-RADS > 2 (Tables 2 and 3; Figs. 3, 4 and 5).

**Table 3:** Sensitivity, specificity, positive predictive value (PPV), and negative predictive value (NPV) for different Node-RADS cutoff values

Node-Rads Cut-off		Youden index (J)	Sensitivity (CI 95%)	Specificity (CI 95%)	NPV (CI 95%)	PPV (CI 95%)	LR+	LR–
Reader- 1	**> 1**	0.685	91.43 (76.9–98.2)	77,08 (62.7–88.0)	32.1 (13.7–58.5)	98.7 (97.8–99.2)	3.99	0.11
	**> 2**	0.538	60.00 (42.1–76.1)	93.75 (92.8–96.7)	11.0 (7.6–15.7)	99.5 (96.3–99.8)	9.60	0.43
	**> 3**	0.457	45.71 (28.8–63.4)	100.0 (92.6–100.0)	8.8 (6.7–11.6)	100.0	NA	0.54
	**> 4**	0.257	25.71 (12.5–43.3)	100.0 (92.6–100.0)	6.6 (5.5–7.9)	100.0	NA	0.74
Reader- 2	**> 1**	0.584	77.14 (59.9–89.6)	81.25 (67.4–91.1)	15.8 (9.1–25.9)	98.7 (97.7–99.3)	4.11	0.28
	**> 2**	0.494	51.43 (34.0–68.6)	97.92 (88.9–99.9)	9.6 (7.0–13.0)	99.8 (98.5–100.0)	24.73	0.50
	**> 3**	0.429	42.86 (26.3–60.6)	100.0 (92.6–100.0)	8.4 (6.5–10.9)	100.0	NA	0.57
	**> 4**	0.143	14.29 (4.8–30.3)	100.0 (92.6–100.0)	5.8 (5.1–6.6)	100.0	NA	0.86

The relationship between the Node-RADS score and lymph node involvement was found to be statistically significant in the χ² test performed for all readers (*p* < 0.001; Table 4).

**Table 4:** *χ*^2^ and linear by linear association

	X^2^ Test	*p* (χ²)	Linear by linear association	*p* (LBLA)
Reader_1	44,940	< 0,001	39,909	< 0,001
Reader_2	31,215	< 0,001	29,043	< 0,001

Comparing the demographic and radiological characteristics of patients with ALNM (+) and ALNM (–), no significant differences were observed between the groups for age, tumor to skin distance, or Ki-67 levels (*p* = 0.713, *p* = 0.729, and *p* = 0.326, respectively). In the ALNM (+) group, the median lymph node size was 8.0 mm (IQR 6.0–11.0), which was significantly larger than in the ALNM (–) group (7.5 mm, IQR 5.0–9.0; *p* = 0.049). Moreover, the mean cortical thickness was significantly higher in the ALNM (+) group compared to the ALNM (–) group (4.88 ± 2.49 mm vs. 3.58 ± 1.21 mm; *p* = 0.004; Table 5).

**Table 5:** Variables associated with axillary lymph node metastasis (ALNM)

	ALNM (-)	ALNM (+)	*p*
**Age** (Mean ± SD)	56.02 ± 15.20	57.17 ± 12.24	0.713*
**Distance from the skin** (Mean ± SD)	15.14 ± 9.20	14.34 ± 11.58	0.729*
**LN Size** (median (Q1-Q3)	7.5 (5.0–9.0)	8.0 (6.0–11.0)	0,049**
**Cortex thickness** (Mean ± SD)	3.58 ± 1.21	4.88 ± 2.49	
**Cortex thickness** (median (Q1-Q3)	3.3 (2.9–3.5)	4.0 (3.5-5.0)	0.004**
**Ki-67 (median (Q1-Q3)**	10.0 (10.0–20.0)	15.0 (10.0–30.0)	0.326**
**ER**			
(-)	11 (%22.9)	8 (%22.9)	0.995^K^
(+)	37 (%77.1)	27 (%77.1)	
**PR**			
(-)	18 (%37.5)	11 (%31.4)	0.567^K^
(+)	30 (%62.5)	24 (%68.6)	
**c-ERB2**			
(-)	34 (%70.8)	20 (%57.1)	0.196^K^
(+)	14 (%29.2)	15 (%42.9)	
**Histologic subtypes of BC**			
DCIS	3 (%6.2)	1 (%2.9)	0.655 ^K^
Invasive Carcinoma	33 (%68.8)	28 (%80.0)	
Lobular Carcinoma	4 (%8.3)	3 (%8.6)	
Mixt type	5 (%10.4)	1 (%2.9)	
Other	3 (%6.2)	2 (%5.7)	
**Family History**			
Negative	14 (%93.3)	20 (%90.9)	0.644^F^
Positive	1 (%6.7)	2 (%9.1)	

*;Student-T test, **; Mann-Whitney U test, ^K;^ Ki-square, ^F;^Fisher’s Exact test

When evaluated in terms of lymph node morphological characteristics, patients with ALNM (+) demonstrated significantly higher rates of round shape (42.9% vs. 4.2%), irregular border (45.7% vs. 2.1%), extranodal extension (54.3% vs. 4.2%), texture heterogeneity (57.1% vs. 8.3%), loss of fatty hilum (52.9% vs. 2.1%), and presence of necrosis (28.6% vs. 0), all of which were statistically significant (*p* < 0.001 for all; Table 6).

**Table 6:** Morphological characteristics associated with axillary lymph node metastasis (ALNM)

	ALNM (-)	ALNM (+)	*p*
Lymph node shape			
Oval/Kidney bean	46 (%95.8)	20 (%57.1)	< 0.001
Round/Spherical	2 (%4.2)	15 (%42.9)	
Lymph node border			
Smooth	47 (%97.9)	19 (%54.3)	< 0.001
Irregular	1 (%2.1)	16 (%45.7)	
Extranodal extension			
(-)	46 (%95.8)	16 (%45.7)	< 0.001
(+)	2 (%4.2)	19 (%54.3)	
Texture			
Homogeneous	44 (%91.7)	15 (%42.9)	< 0.001
Heterogeneous	4 (%8.3)	20 (%57.1)	
Fatty hilum			
(-)	1 (%2.1)	18 (%52.9)	< 0.001
(+)	47 (%97.9)	16 (%47.1)	
Necrosis			
(-)	48 (%100.0)	25 (%71.4)	< 0.001
(+)	0 (%0.0)	10 (%28.6)	

Regarding the results of correlation analysis between variables, significant positive correlation was found between Node-RADS and lymph node size by Reader 1 and Reader 2 (*r* = 0.579; *p* < 0.001, *r* = 0.546; *p* < 0.001, respectively). Similarly, there was a strong positive correlation between Node-RADS and cortical thickness found by Reader 1 and Reader 2 (*r* = 0.541; *p* < 0.001, *r* = 0.480; *p* < 0.001, respectively).

**Fig. 6:**
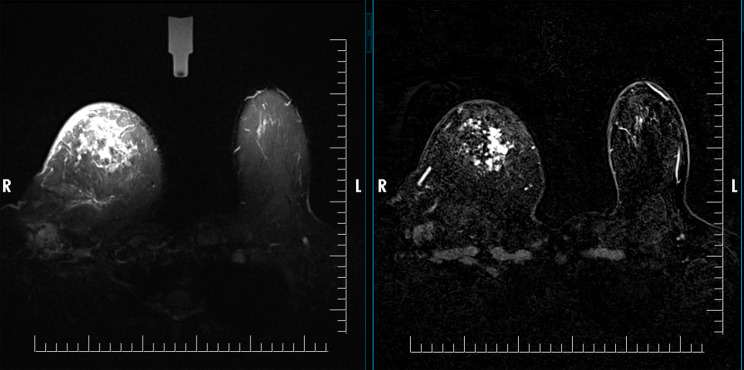
On axial STIR (left) and Dixon (right), a lymph node in the right axilla demonstrates features of extranodal extension, with heterogeneous internal texture and no identifiable fatty hilum, corresponding to a Node-RADS category 5

In the logistic regression analysis, a fatty hilum was a statistically significant predictor of lymph node status (*p* = 0.001). Specifically, nodes with a preserved fatty hilum demonstrated a markedly lower likelihood of metastasis (OR = 0.012, 95% CI: 0.001–0.154), indicating that the absence of a fatty hilum strongly correlates with metastatic involvement. Flare sign was statistically associated with lymph node metastasis (*p* = 0.013, OR = 11.78, 95% CI: 1.69–81.91; Fig. 6). This finding indicates that patients with the flare sign had approximately a ninefold higher risk of lymph node metastasis. The internal texture of the lymph node was also found to be statistically significantly associated with invasion (*p* = 0.002, OR = 0.015, 95% CI: 0.001–0.162). Lymph node border, cortical thickness, shape and necrosis were not found to be significantly associated with an increased risk of ALN involvement (*p* > 0.05; Table 7).

**Table 7:** Logistic regression analysis

	*p*	Exp(B)	95% C.I.for EXP(B)	
			Lower	Upper
Cortical thickness	0.719	1.130	0.581	2.200
Lymph node shape	0.506	0.318	0.011	9.275
Lymph node border	0.065	23.834	0.823	690.48
Extranodal extension	0.013	11.78	1.696	81.910
Fatty hilum	0.001	0.012	0.001	0.154
Texture	0.002	0.015	0.001	0,162

When inter-reader agreement for Node-RADS scoring was evaluated, a high level of concordance was observed between Reader 1 and Reader 2 (κ = 0.814), demonstrating strong interobserver reliability and supporting the consistent application of Node-RADS across different observers.

## Discussion

The meta-analysis by Le Boulc’h et al. evaluated the diagnostic accuracy of ultrasound (US), MRI, and FDG-PET for detecting ALN metastases in breast cancer patients. The study demonstrated that the US possesses exceptionally high specificity (100%) but relatively low sensitivity (51%), while MRI offers a more balanced performance (sensitivity 83%, specificity 85%). FDG-PET, although highly specific (94%), exhibited limited sensitivity (49%). Collectively, these results indicate that no single imaging modality can provide sufficiently reliable axillary staging in clinical practice [17]. According to the scientific findings reported by Aktaş et al. (2021), even when US, MRI, and PET/CT yielded negative results, approximately 14.3% of cases were false-negative for axillary metastasis, particularly in small metastatic foci [18]. These discrepancies highlight the inherent limitations of imaging techniques, including inter-observer variability. In the current study, the interobserver variability values demonstrated a high level of agreement, suggesting that MRI-based ALN scoring, like Node-RADS, may offer superior reproducibility and diagnostic reliability in this context. However, a standard score for evaluation with US and CT has not yet been created.

Lymph nodes that appear markedly enlarged and exhibit abnormal morphology, particularly when their appearance differs significantly from other axillary nodes, are highly indicative of metastatic involvement. Characteristic morphologic alterations associated with metastasis include cortical thickening, loss of the fatty hilum, and a rounded shape or a long-to-short axis ratio less than two [19]. Furthermore, irregular nodal margins and a heterogeneous cortical signal observed on MRI have been reported to demonstrate high specificity for metastatic disease [20]. In the current study, lymph node shape, margins, the flare sign, loss of the fatty hilum, internal texture, and necrosis were found to be significantly associated with metastasis, consistent with previous literature. Moreover, the observation that including multiple such criteria in Node-RADS scoring enhances its diagnostic accuracy and predictive performance suggests that these combined morphologic features play a pivotal role in improving overall staging reliability. Although metastatic ALNs are traditionally considered enlarged when the short-axis diameter exceeds 10 mm, our findings showed that many metastatic nodes measured below this threshold. This underscores the limitation of relying solely on size-based criteria in axillary staging. To clarify this discrepancy, we added a comparison with the conventional > 10 mm standard, emphasizing that metastatic involvement frequently occurs in non-enlarged lymph. Thus, size alone may not be a sufficient discriminator and additional morphologic features, such as cortical thickening or flare sign indicative of extranodal extension, should be integrated into radiologic assessment. Consistent with the observations of Sanlı et al. [21], this suggests that the size threshold for axillary evaluation may require reconsideration. Moreover, even in enlarged nodes, the presence of a fatty hilum seems to indicate non-metastatic. Collectively, these findings support the need for a breast-specific refinement of Node-RADS, integrating morphologic and diffusion-weighted MRI parameters to improve diagnostic accuracy and reproducibility. In addition, radiologic evaluation and visualization of the flare sign in extranodal extension suggest that this feature could be considered an additional parameter within the scoring system. Previous studies have described several imaging signs associated with extranodal extension, including flare sign, which has been reported to demonstrate high specificity for identifying metastatic lymph nodes [22, 23]. In our analysis, because flare signs were used concurrently to detect extranodal extension, we found that their presence increased the risk of metastasis approximately nine-fold, underscoring their potential diagnostic significance in ALN assessment. Consistent with the findings of Pediconi et al. and based on the balanced sensitivity and specificity values across all readers, we determined that a Node-RADS score greater than 2 is the optimal cut-off for predicting lymph node involvement [14]. This threshold effectively differentiates between suspicious and non-suspicious lymph nodes, supporting its clinical applicability in axillary staging. Both readers demonstrated high consistency, with agreement ranging from good to excellent. In most instances, analysis of Node-RADS cut-off subclasses revealed even stronger alignment, suggesting that the system can be applied reliably and effectively in clinical practice. When comparing sensitivity and specificity values between the two studies, the results of the current study demonstrate that sensitivity was consistently lower across all Node-RADS scores. In contrast, specificity was relatively higher, particularly at lower score thresholds. This pattern suggests that while our scoring approach may yield fewer false positives, it may under-detect certain cases of nodal metastasis, underscoring the balance between diagnostic sensitivity and specificity in axillary staging.

The performance metrics observed in this study demonstrated notable differences compared with the larger cohort analyzed by Kim et al. In their series, a Node-RADS cutoff of ≥ 3 yielded sensitivities of 71.1% for invasive carcinoma (IDC) and 52.5% for invasive lobular carcinoma (ILC), with specificities of 86.5% and 85.1%, respectively. In contrast, at a comparable cutoff (> 3), our study showed a specificity of 100% for both readers, while sensitivities were lower (45.7% for Reader 1 and 42.8% for Reader 2). Similarly, when evaluating the > 2 threshold, our findings again revealed lower sensitivity (60% for Reader 1; 51% for Reader 2) but higher specificity (93.7% for Reader 1; 97.9% for Reader 2) relative to the balance reported by Kim et al. [15]. Kim et al. analyzed a substantially larger population and performed separate evaluations for IDC and ILC. In contrast, the present study included a limited number of ILC cases, precluding subtype-specific analysis. This difference in cohort size and histologic composition may have contributed to the lower sensitivity observed in our study, as reduced sample size and limited histologic diversity can restrict statistical power and hinder accurate assessment of subtype-specific diagnostic performance. In addition, variations in reader experience may have influenced diagnostic sensitivity, particularly for subtle morphologic features assessed within the Node-RADS framework. Differences in patient composition, tumor histology distribution, and disease prevalence and imaging protocols are known to influence diagnostic thresholds and may partially explain why a cutoff of ≥ 3 was optimal in Kim et al.’s cohort, while > 2 provided the most balanced diagnostic performance in the current study. Despite these differences, both studies consistently identified Node-RADS as an independent predictor of ALN metastasis, supporting its clinical utility in breast cancer staging. It should be acknowledged that standard breast MRI protocols are primarily optimized for the evaluation of breast parenchyma; thus, the field of view is not routinely designed to ensure complete coverage of the entire ALN chain. As a result, axillary assessment particularly of level II and III lymph nodes may be incomplete in routine clinical practice. This inherent technical limitation of breast-specific MRI protocols may partially affect the sensitivity of MRI-based axillary staging and should be considered when interpreting the clinical performance of Node-RADS.

This study has several limitations. First, the research was conducted as a single-center, retrospective analysis with a relatively small sample size, which inherently restricts the statistical power of subgroup evaluations. Although the cohort included different histologic subtypes, the number of cases within some subgroups was too limited to allow reliable stratified analysis. As a result, we were unable to perform detailed subtype-specific evaluations. Larger, prospectively designed, multi-center cohorts would validate our findings and enhance their generalizability.

## Conclusions

In conclusion, clearly defining the clinical implications of specific Node-RADS categories may help guide decision-making in axillary management. A score below 2 is generally associated with a very low probability of nodal metastasis and may therefore reduce the need for unnecessary biopsy or surgical intervention, whereas scores of 3 or higher indicate increasing suspicion and warrant targeted ultrasound assessment or image-guided biopsy. Incorporating such score-based stratification into routine practice may contribute to more selective use of axillary procedures and minimize avoidable morbidity. However, size and morphologic features notably cortical thickness and flare sign demonstrated a meaningful association with metastatic involvement. Rather than proposing these findings as replacements for existing Node-RADS criteria, they should be considered as potential supplementary markers to enhance diagnostic confidence when used alongside the current scoring components. Further studies are needed to clarify how these parameters could be integrated into the system in a standardized manner. In addition, incorporating a short-axis diameter threshold of less than 10 mm, which is considered a critical criterion, into Node-RADS scoring should be validated using large, multicenter datasets before being adopted as a standardized parameter.

## Data Availability

Additional information about the methodology and implementation is available upon reasonable request from the corresponding author.
